# Bio-Mimicking Acellular Wet Electrospun Scaffolds Promote Accelerated Integration and Re-Epithelialization of Full-Thickness Dermal Wounds

**DOI:** 10.3390/bioengineering9070324

**Published:** 2022-07-18

**Authors:** Jiah Shin Chin, Leigh E. Madden, Anthony R. J. Phillips, Sing Yian Chew, David L. Becker

**Affiliations:** 1 Nanyang Institute of Health Technologies, Interdisciplinary Graduate School, Nanyang Technological University, Singapore 639798, Singapore; chin_jiah_shin@asrl.a-star.edu.sg; 2 School of Chemical and Biomedical Engineering, Nanyang Technological University, Singapore 637459, Singapore; 3 Lee Kong Chian School of Medicine, Nanyang Technological University, Singapore 308232, Singapore; lmadden@ntu.edu.sg; 4 Department of Surgery, School of Biological Sciences, University of Auckland, Auckland 1010, New Zealand; a.phillips@auckland.ac.nz; 5 School of Materials Science and Engineering, Nanyang Technological University, Singapore 639798, Singapore; 6 Skin Research Institute Singapore, Clinical Sciences Building, 11 Mandalay Road, Singapore 308232, Singapore

**Keywords:** electrospinning, wound healing, skin regeneration, collagen, fiber scaffolds, polycaprolactone

## Abstract

Scaffolds can promote the healing of burns and chronic skin wounds but to date have suffered from issues with achieving full skin integration. Here, we characterise the wound response by both tissue integration and re-epithelialization to a scaffold using wet electrospinning to fabricate 3D fibrous structures. Two scaffold materials were investigated: poly(ε-caprolactone) (PCL) and PCL + 20% rat tail type 1 collagen (PCL/Coll). We assessed re-epithelisation, inflammatory responses, angiogenesis and the formation of new extracellular matrix (ECM) within the scaffolds in rat acute wounds. The 3D PCL/Coll scaffolds impeded wound re-epithelisation, inducing a thickening of wound-edge epidermis as opposed to a thin tongue of migratory keratinocytes as seen when 3D PCL scaffolds were implanted in the wounds. A significant inflammatory response was observed with 3D PCL/Coll scaffolds but not with 3D PCL scaffolds. Enhanced fibroblast migration and angiogenesis into 3D PCL scaffolds was observed with a significant deposition of new ECM. We observed that this deposition of new ECM within the scaffold was key to enabling re-epithelialization over the scaffold. Such scaffolds provide a biocompatible environment for cell integration to lay down new ECM and encourage re-epithelisation over the implanted scaffold.

## 1. Introduction

Chronic wounds such as diabetic foot ulcers and venous leg ulcers typically fail to heal in an orderly and timely manner [1]. The prevalence of chronic wounds continues to be a serious and growing problem worldwide and is reaching epidemic proportions [1,2,3]. The yearly health care costs from chronic wounds in the United States of America is estimated to already be USD 28 billion to 96.8 billion [4] and will become an increasingly significant issue as our global population continues to grow older and more obese.

The treatment of chronic wounds is a complex challenge. Commercial wound dressings protect the wound bed while providing a favorable environment to encourage wound healing [5]. However, in chronic wounds, the dermis is severely damaged, and these dressings are insufficient to help replace lost tissue. Scaffolds can serve as artificial dermal matrix substitutes to encourage repair of these wounds. Beyond providing structural support for cells, scaffolds can also function as bioactive factors and therapeutic reservoirs [6]. Previously, it has been reported that electrospun scaffolds functionalized with antisense oligodeoxynucleotides (asODNs) targeting the mRNA of gap junction protein connexin 43 (Cx43) accelerated wound healing and dampened inflammatory responses [7]. Although sustained release of Cx43asODN was achieved with this scaffold delivery platform, re-epithelialization failed to occur over the scaffolds [8]. It was shown that the epithelium grew under the scaffold as if it were a scab and so failed to integrate the scaffold into the wound.

In this current study, we adapted a wet electrospinning fabrication method to produce three-dimensional (3D) scaffolds that better mimic the native extracellular matrix (ECM) of the dermis. Unlike conventional electrospinning that produces fiber sheets, wet electrospinning is a simple yet effective method to produce 3D, sponge-like matrices without the use of chemical additives and sophisticated devices [9]. Here, a liquid collector, instead of a metallic solid collector, is used. The liquid collector rapidly solidifies the polymer solution jet, resulting in the formation of instantaneously solid structures that upon drying, appear bulky and fluffy.

We hypothesized that these wet electrospun scaffolds could integrate better by enhancing fibroblast migration and new ECM deposition into the scaffolds, then encourage re-epithelialization over these scaffolds in full-thickness wounds.

In this work, we examined the microscopic healing response in the presence of these wet electrospun scaffolds with particular focus on the wound-edge outgrowth, inflammatory response and new ECM deposition within these scaffolds. Two materials were explored—Poly(ε-caprolactone) (PCL) and PCL with 20% rat tail type 1 collagen. Collagen was added to better mimic the ECM of the dermis and improve the biocompatibility of scaffolds. We also evaluated the structural stability of these scaffolds to ensure that the use of these scaffolds in chronic wound bed is compatible with existing clinical wound care practices. We found full integration and skin re-epithelisation could be achieved with this new scaffold.

## 2. Experimental Section

### 2.1. Materials

Poly(ε-caprolactone) (PCL, Sigma, Mw: 80 kDA), 2,2,2-trifluoroethanol (TFE), dimethylformamide (DMF), F127 pluronic gel, paraformaldehyde, sucrose, concentrated hydrochloric acid, sodium carbonate, magnesium sulfate, limonene and acetic acid were purchased from Sigma-Aldrich. The 1 mL Luer lock syringe and 22G needles were purchased from Becton, Dickinson and Company (BD). Absolute ethanol was purchased from Merck. The 96-well plates and rat tail type 1 collagen were purchased from Corning. Nair^®^ depilatory cream was purchased from Church and Dwight. Commercial-grade 100% ethanol was purchased from Aik Moh paints and chemicals and diluted to 2 other concentrations—70% and 95%—with distilled water. The 6 mm biopsy punches were purchased from Integra Miltex. Tegaderm™ film was purchased from 3M. OPSITE was purchased from Smith and Nephews. Phosphate-buffered saline was purchased at 10× concentration from Bio Basic and diluted to 1x concentration for use in this study (PBS). Tissue-Tek OCT compound was purchased from Sakura Finetek. Clearene, haematoxylin and alcoholic eosin Y 515 were purchased from Leica Biosystems. Citifluor AF-1 was purchased from Electron Microscopy Science. Picrosirius red was purchased from Abcam.

### 2.2. Fabrication of 3D Scaffolds

PCL was first dissolved at 14% (*w*/*w*) in a mixture of TFE and DMF at 4:1 (*v*/*v*). The dissolved PCL solution was subsequently loaded into a 1 mL Luer lock syringe and capped with a 22 G blunt needle before placing on an infusion pump (KD Scientific, KD-100, Holliston, MA, USA). The infusion pump was mounted onto aluminium profiles to allow the vertical deposition of polymer solution into the collector. A metal container (inner diameter ϕ = 11.8 cm, outer diameter ϕ = 12.2 cm) was then placed directly below the infusion pump and 14.6 cm away from the needle tip (Supplementary Figure S1A). This metal container was filled with 100 ml of absolute ethanol to achieve a 2.5 cm height ethanol bath, where PCL fibers were eventually collected. The 3D PCL scaffolds were electrospun at a flow rate of 8 ml/h and voltages of +8 and −4 kV were applied to the needle tip and the metal container, respectively. A volume of 7 µL of polymer solution was spun into the ethanol bath and the electrospun fibers were moulded into shape (height = 1.5 mm, diameter ϕ = 6 mm) by placing these fibers into a 96-well plate. The fibers were then lyophilised overnight to produce 3D PCL scaffolds that match the size and shape of the excisional wounds.

To fabricate collagen-containing scaffolds, PCL and rat tail type 1 collagen were mixed at a 20:1 weight ratio, respectively, before dissolving the polymer blend in a mixture of TFE and DMF at 4:1 (*v*/*v*). Upon complete dissolution, the polymer solution was loaded into a 1 mL Luer lock syringe and placed onto the infusion pump. An identical setup, as mentioned above, was used to fabricate these collagen-containing scaffolds. However, the metal container was filled with 75 mL of absolute ethanol instead to achieve a 2 cm height ethanol bath for the collection of electrospun fibers. Similarly, 7 µL of the blended polymer solution was electrospun and collected into the ethanol bath. Fibers were placed into a 96-well plate and lyophilised overnight to produce 3D PCL/Coll scaffolds that were customised to conform to the excisional wounds.

### 2.3. Physical Characterisation of Scaffolds

The morphology of both 3D PCL and 3D PCL/Coll scaffolds was evaluated by scanning electron microscopy (SEM) (JOEL, JSM-6390LA, Akishima, Tokyo, Japan) under an accelerating voltage of 10 kV after sputter-coating with platinum at 10 mA for 100 s. To quantify the average fiber diameters of these scaffolds, 100 fibers from each technical repeat were measured using Image J’s measure tool [10] (National Institutes of Health, Bethesda, MD, USA). Similarly, the average pore size of these scaffolds was quantified by measuring 100 pores from each technical repeat with Image J’s measure tool [10]. A total of 3 technical repeats for each scaffold were conducted for both fiber diameter and pore size quantification.

### 2.4. In Vivo Studies

#### 2.4.1. Rodent Strains

All animal procedures were approved and performed in accordance with the Institutional Animal Care and Use Committee (IACUC) in Nanyang Technological University (IACUC Protocol A0367). A total of 12 male Sprague Dawley rats (250 to 300 g) were obtained from InVivos Pte. Ltd., Singapore, and used in experiments at 6 weeks old. All rats had access to food and water. In addition, they were housed in litter cages and were exposed to a 12 h light/dark cycle in the housing room. All animals were weighed prior to surgery to calculate drug dose.

#### 2.4.2. In Vivo Full-Thickness Wounding

Anaesthesia was induced using 4% isoflurane and maintained at 2% during the whole surgical procedure. Animals were injected subcutaneously with 0.03 mg/mL buprenorphine as analgesia before wounding. The backs of the rats were carefully shaved with an electric hair trimmer before applying a thin layer of Nair^®^ depilatory cream onto the shaved skin for 2 min. Thereafter, both the cream and fur were removed using a warm moistened gauze pad. Once the prepared skin was completely bare, the animals were transferred onto a heated mat for the duration of the surgical procedure.

Prior to wounding, the bare skin was wiped with 70% ethanol three times. Chlorohexidine and iodine were not used as they can interfere with the normal skin healing process. Dorsal skin was first tented, parallel to the spine, prior to carefully making full-thickness excisional wounds on the backs of these animals with a standardised 6 mm biopsy punch (Supplementary Figure S1B). A total of 4 wounds were made on the backs of each rat. Scaffolds wetted with 20 μL of 30% (*w*/*v*) F127 pluronic gel or F127 pluronic gel alone as control were applied directly into these excisional wounds before covering each wound individually with small sections of Tegaderm™ film. F127 pluronic gel was adopted as the control as it fills the excision wound and is known for encouraging healing even in the absence of therapeutics [11]. Notably, both saline and PBS cannot be directly applied on the full-thickness wound—solutions leaked out upon dressing the wounds with Tegaderm. In contrast, pluronic gel sets at room temperature and remains in the wound bed. The solution to wet the scaffolds must also be applicable to the wound independently of the scaffold to establish a fair control. Hence, pluronic gel was chosen to wet the scaffolds instead. Placement of scaffolds and F127 pluronic gel was randomised from rat to rat. The entire dorsum area was dressed with a single piece of OPSITE to ensure that the wounds remain clean while holding the wound contents in place. Once all surgical procedures were completed, all animals were kept in a heated chamber and monitored for recovery. Once recovered, animals were re-housed in individual cages and monitored regularly.

#### 2.4.3. Wound Harvesting

Animals were euthanised via carbon dioxide inhalation and cervical dislocation was used as a secondary means to assure death—a humane sacrificial method endorsed by the IACUC. Animals were culled at 1, 3 or 5 days post wounding (n = 4 at each time point). A large sheet of back skin containing the wounds was excised and spread onto smooth waxed carboard to prevent the skin from curling during fixation. Wounds were subsequently individually excised, bisected and fixed by immersion in a 4% paraformaldehyde solution for 24 h at 4 °C.

### 2.5. Histology Procedures

The first half of each wound was washed 3 times in phosphate-buffered saline and then transferred to 20% sucrose solution for 24 h at 4 °C. These cryo-protected tissues were then embedded and snap frozen in Tissue-Tek OCT compound. Subsequently, these embedded tissues were sectioned using a cryostat (Leica Biosystems, CM1900, Nussloch, Germany) at a thickness of 8 μm. These sections were used to understand scaffold degradation across the different harvest timepoints. The other half of each wound was washed once in 70% ethanol before dehydrating in 70% ethanol for 24 h at 4 °C. Tissues were subsequently transferred to a tissue processor (Leica Biosystems, HistoCore PEARL, Nussloch, Germany) for further processing. Processed tissues were then paraffin wax embedded and sectioned at 6 μm using a rotary microtome (Leica Biosystems, RM2245, Germany).

### 2.6. Haematoxylin and Eosin (H&E) Staining

All staining was performed with an autostainner (Leica Biosystems, ST5010 Autostainner XL, Germany). Slides with 6 μm thick paraffin wax sections were submerged for 5 min in a container of Clearene and changed into another container of Clearene for 5 min to dewax these tissues. Sections were then immersed in 100% ethanol for 2 min, a second container of 100% ethanol for 2 min, 95% ethanol for 2 min, a second container of 95% ethanol for 2 min, 70% ethanol for 2 min and rinsed in a container of running tap water for 2 min to rehydrate the tissues. Thereafter, sections were submerged in haematoxylin for 1 min and rinsed in a container of running tap water for 3 s to remove excess dye. Sections were then immersed in 0.3% acid alcohol (0.3% (*v*/*v*) concentrated hydrochloric acid, 70% (*v*/*v*) ethanol and 29.97% (*v*/*v*) distilled water) and further rinsed in running tap water for 2 min to remove any background staining. To enhance the contrast of the haematoxylin stain, sections were dipped into Scott’s tap water (0.5% (*w*/*v*) sodium bicarbonate and 5% (*w*/*v*) magnesium sulphate dissolved in distilled water) for 5 s before rinsing the sections again with running tap water for 2 min. Haematoxylin-stained sections were then immersed into alcoholic eosin Y 515 for 2 min. Subsequently, sections were submerged in 70% ethanol for 2 min, 95% ethanol for 2 min, a second container of 95% ethanol for 2 min, 100% ethanol for 2 min, a second container of 100% ethanol for another 2 min and Clearene for 2 min to dehydrate the sections. Slides with stained sections were then individually removed, covered with 1 drop of limonene and then sealed using a coverslip. The following histological observations were performed by 3 operators blinded to the experimental setup. All 3 operators have been trained to analyse H&E skin sections.

### 2.7. Nascent Epidermal Thickness Measurements

Images of H&E-stained sections were obtained using a slide scanner (Carl Zeiss, Zeiss Axioscan Z1, Oberkochen, Germany). Tile scan images of these sections were captured with a 20× objective. Whole-tissue sections were analysed using Zeiss Zen Black software (Carl Zeiss, Germany), where regions of interest (1000 µm by 1000 µm) were exported for further analysis in Image J [10] (Measure tool). The thickness of the epidermis at the wound edge was determined by measuring the thickest point along the axis from the basal to spinous layer within 150 μm of the nascent epidermal leading edge. This measurement was performed at both wound edges and averaged to quantify the thickness of nascent epidermis for each sample.

### 2.8. Polymorphonuclear Leukocyte and Macrophage Quantification

The extent of inflammation within and around the scaffolds was evaluated based on the polymorphonuclear leukocyte (PMN) and macrophage infiltration. Regions of interest (200 µm by 200 µm) from tile scan images of the H&E-stained sections were exported using Zeiss Zen Black software for further quantification of PMNs and macrophages using Image J [10] (Cell Counter). To evaluate the extent of inflammation with the scaffolds, 2 regions of interest were exported. These were selected from the host–implant interface. Similarly, to evaluate the extent of inflammation around the scaffolds, 4 regions of interest that were 700 µm away from the wound edge were exported for the quantification.

### 2.9. Assessment of Scaffold Structural Integrity

The 8 μm thick frozen sections were allowed to thaw at room temperature for 15 min before using PBS to rinse away any residual OCT compound on the tissues. Sections were then covered with a drop of Citifluor AF-1 and mounted with a coverslip.

Electrospinning generally resulted in birefringent fibers [12]. Polarized light microscopy was adopted here to examine the presence of electrospun scaffolds within the wound bed. Images were captured at 10x magnification using a polarized light microscope (Leica, DMi8, Germany) and automatically stitched using Leica LAS X software (Leica, Germany) to obtain tile scan images of the tissue sections. Tile scan images were analysed using Zeiss Zen Black software, where 4 regions of interest (500 µm by 500 µm) were exported out for further analysis in Image J [10]. The pixel area of each region of interest was measured using Image J [10] (Particle Analysis) and the values of all 4 regions of interest were averaged to arrive at an average pixel area per field of view for the tissue section. At least 3 sister sections were analysed at each time point for each wound to determine the extent of scaffold degradation. Representative high-power images within these scaffolds were captured with a 20× objective using the polarized light microscope.

### 2.10. Fibroblast and Blood Vessel Quantification

The proliferative phase of wound healing was evaluated based on the extent of fibroblast migration into the scaffolds and the formation of blood vessels within the scaffolds. Three regions of interest (350 µm by 350 µm) from tile scan images of the H&E-stained sections were exported using Zeiss Zen Black software for further quantification of fibroblasts and blood vessels using Image J [10] (Cell Counter).

### 2.11. Quantification of Collagen Deposition in Implanted Scaffolds by Picrosirius Red Staining

The quantification of collagen deposition on the fibers of the scaffolds was performed based on an established protocol [13] with slight modifications. Briefly, 6 μm thick paraffin wax sections were stained with picrosirius red dye for histological visualization of collagen fibers. Prior to staining, these sections were dewaxed and rehydrated with protocols mentioned under “Haematoxylin and Eosin Staining”. Sections were then submerged in haematoxylin for 5 min and rinsed in a container of running tap water for 5 min to remove excess dye. Subsequently, sections were submerged into picrosirius red for 1 h. To remove excess dye and preserve the stains, sections were dipped into 2 containers of 0.5% acetic acid, each for 5 s. The stained sections were further dehydrated in 2 changes of 100% ethanol for 5 s each before covering each section with 1 drop of limonene and then sealed with a coverslip.

To evaluate the extent of collagen deposition within the scaffolds, these picrosirius red-stained sections were examined under 63x magnification using a polarized light microscope (Leica, DMi8, Germany). Brightfield images were simultaneously acquired to provide locality of regions of interest. All polarized microscopy images in the wound were overexposed (compared to intact skin) to reveal the fine strands of new collagen fibers deposited within the scaffolds. There were 8 regions of interest within the implanted scaffolds were imaged (4 taken at the center of scaffold and 4 taken at the edges of the scaffold). Acquired images were further processed with Image J [10]. All images were split into 3 channels—red, green and blue. Mean grey value measurement was conducted on both green and red channel images (Image J’s Particle Analysis [10]). New collagen fibers, stained with picrosirius red followed by polarizing microscopy, are known to be weakly birefringent—appearing as greenish or reddish fibers (depending on the angle of imaging) [14]. The mean grey values from the respective regions of interest were averaged to determine the extent of collagen deposition within the scaffold.

### 2.12. Re-Epithelialization Distance Measurement

The length of nascent epidermis outgrowth from the wound edge was measured on tile scan images of the H&E-stained sections. Regions of interest (2000 µm by 2000 µm) were exported from these images using Zeiss Zen Black software. Measurement of outgrowth was conducted at both wound edges using Image J [10] (Measure tool) and averaged to arrival at a re-epithelialization distance measurement for each sample.

### 2.13. Statistical Analysis

Statistical analysis was carried out using GraphPad Prism software. All results were presented as the mean ± standard deviation. The Brown–Forsythe test was used to test for equal variances while the Shapiro–Wilk test was used to test for normality. If the variances of the groups were equal and normally distributed, one-way ANOVA and Tukey’s post hoc tests were used to compare the means of more than two samples. Where only 2 samples were compared, Student’s t-test was used. Where variances were not equal, the non-parametric tests, Kruskal–Wallis and Mann–Whitney tests, were used.

## 3. Results

### 3.1. Fabrication of 3D Scaffolds

**Figure 1:**
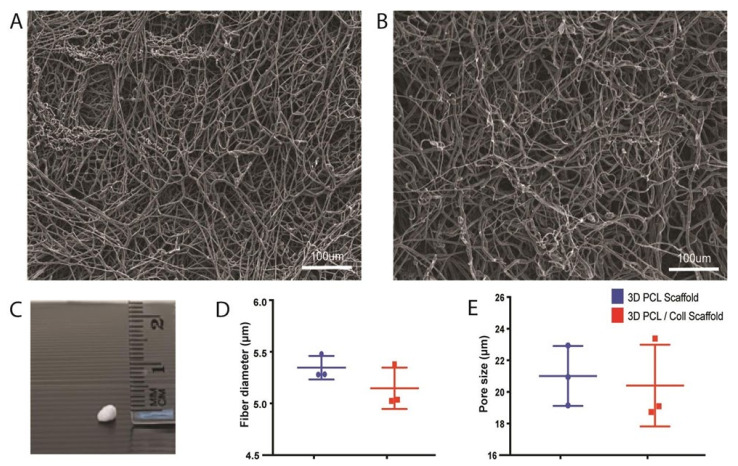
Physical characterisation of wet electrospun scaffolds. (**A**) SEM of PCL scaffolds. Scale bar represented by white line for 100 µm. (**B**) SEM of PCL/Collagen scaffolds. Scale bar represented by white line for 100 µm. (**C**) Digital photograph of a wet electrospun scaffold. (**D**) Quantification of fiber diameter. Mann–Whitney test (n = 3, where n represents 1 scaffold technical repeat). No significant difference between both scaffolds (*p* = 0.4). (**E**) Quantification of pore size. Mann–Whitney test (n = 3, where n represents 1 scaffold technical repeat). No significant difference between both scaffolds (*p* = 0.7).

To understand the physical characteristics of these wet electrospun scaffolds, SEM was performed, and digital images were captured for subsequent analysis (Figure 1). Both 3D PCL scaffolds and 3D PCL/Coll scaffolds displayed porous structures (Figure 1A,B, respectively). Macroscopically, these scaffolds resembled “cotton balls” and were of 1–1.5 mm in thickness (Figure 1C). Detailed analysis of the SEM images revealed that 3D PCL scaffolds have an average fiber diameter of 5.35 ± 0.114 µm and an average pore size of 21.0 ± 1.89 µm. Similarly, 3D PCL/Coll scaffolds were found to have an average fiber diameter of 5.15 ± 0.200 µm and an average pore size of 20.4 ± 2.59 µm. Although variation in fiber diameter and pore size were observed within each type of scaffold, no significant difference was observed between and within these materials (Figure 1D,E).

### 3.2. Aberrant Thickening of Wound-Edge Epidermis Induced by 3D PCL/Coll Scaffolds

**Figure 2:**
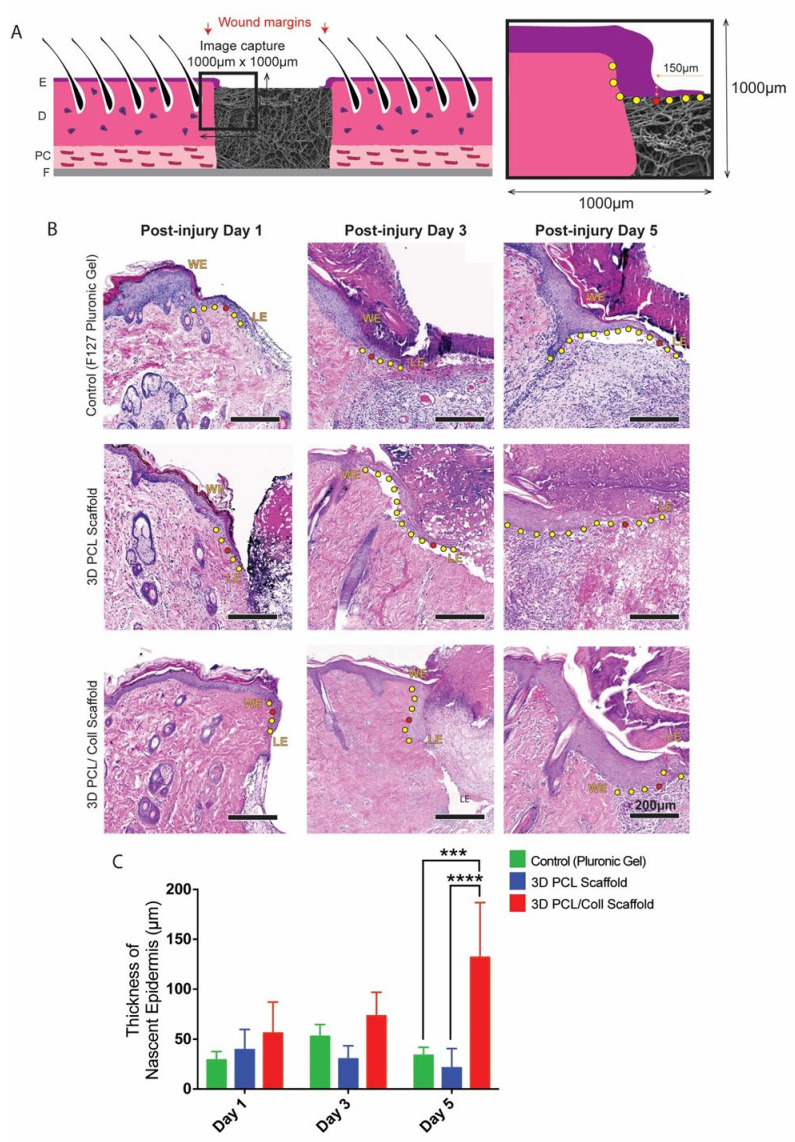
(**A**) Schematic of tissue section. An enlarged schematic is shown on the right to depict measurement of epithelial tongue thickness. The red dot indicates the point where nascent epidermis thickness was measured (i.e., 150 µm away from tip of epithelial tongue. The yellow dots trace the outgrowth of epithelial tongue from wound edge. E: epidermis. D: dermis. PC: panniculus carnosus. F: facia. (**B**) H&E images of wound edges. WE: wound edge. LE: leading edge. Scale bar represented by black line for 200 µm. (**C**) Quantification of nascent epidermis thickness. One-way ANOVA; Tukey’s post hoc test (n = 4, where n represents 1 biological repeat). ***: *p* < 0.001, ****: *p* < 0.0001.

To assess whether scaffolds were promoting wound healing, histological examination was conducted on excised wounds. In particular, the epithelial tongue at the wound edges were examined. F127 pluronic gel was applied to one of the wounds as a control for the scaffolds. At day 1 post injury (D1), no significant difference in nascent epidermal tongue thickness was observed. However, at day 3 (D3) and day 5 post injury (D5), keratinocytes in the wound edges of 3D PCL/Coll scaffold-treated wounds formed thickened, non-migratory bulbs compared to the thin tongues of cells that were formed in the rapidly healing control wounds and 3D PCL scaffold-treated wounds (Figure 2B). In particular, at D5, the average thickness of nascent epidermis of 3D PCL/Coll-treated wounds was 131 ± 54.8 µm—significantly thicker than the control and 3D PCL scaffold-treated wounds (*p* < 0.001 and *p* < 0.0001, respectively, Figure 2C).

### 3.3. Aggressive Foreign Body Reaction and Inflammatory Response towards 3D PCL/Coll Scaffolds

**Figure 3:**
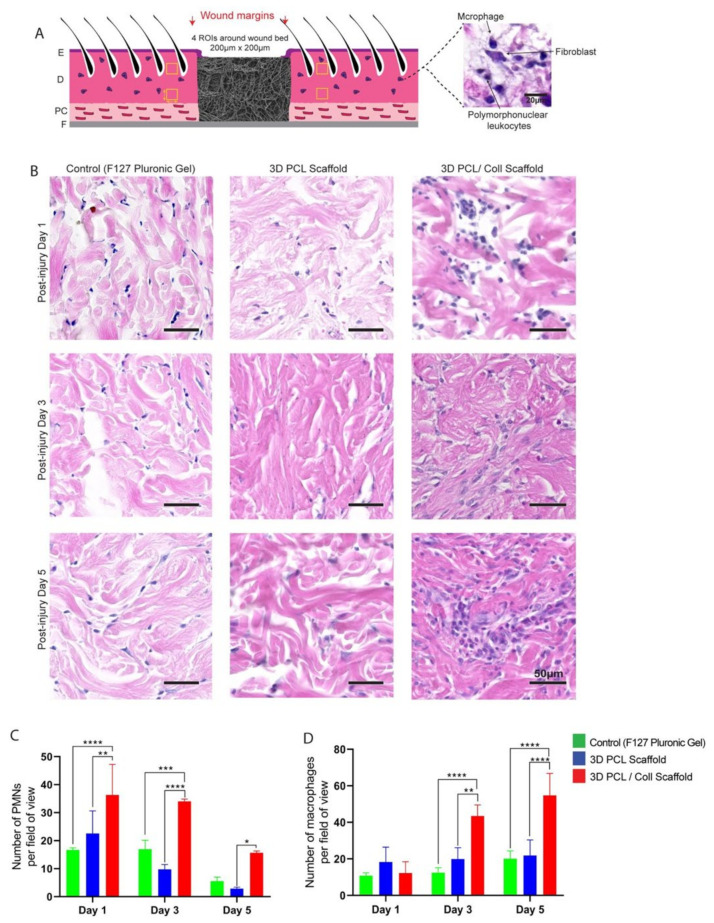
(**A**) Schematic of tissue section, where four regions of interest were cropped for quantification. E: epidermis. D: dermis. PC: panniculus carnosus. F: facia. (**B**) Representative H&E images of dermis 700 µm away from wounds edge at post injury days 1, 3 and 5. Wounds were treated either with F127 pluronic gel, 3D PCL scaffold or 3D PCL/Coll scaffold. Scale bar represented by black line for 50 µm. (**C**) Quantification of dermal PMNs in dermis near wound edge. One-way ANOVA; Tukey’s post hoc test (n = 4, where n represents 1 biological repeat). *: *p* < 0.5, **: *p* < 0.01, ***: *p* < 0.001, ****: *p* < 0.0001. (**D**) Quantification of macrophages in dermis near wound edge. One-way ANOVA; Tukey’s post hoc test (n = 4, where n represents 1 biological repeat). **: *p* < 0.01, ****: *p* < 0.0001.

Polymorphonuclear leukocytes (PMNs) and macrophages were identified by their distinctive morphologies and counted in the intact dermis distal to the wound edge to investigate the inflammatory response towards the scaffolds (Figure 3B). At D1, wounds treated with 3D PCL/Coll scaffolds generated the highest PMN count with an average of 33 ± 14 PMN per field of view followed by wounds treated with 3D PCL scaffolds (22 ± 7, *p* < 0.001) and control wounds (16 ± 1, *p* < 0.001). At D3, there was a slight decrease in PMN counts across all three treatments. However, wounds treated with 3D PCL/Coll scaffolds were consistently found to possess the highest PMN count even up to D5 when compared across all treatment groups. Interestingly, wounds treated with 3D PCL scaffolds had the lowest PMN count at D5 (2, *p* < 0.05, Figure 3C). Following the clearance of PMNs from these wounds, a significant increase in macrophage count was observed for wounds treated with 3D PCL/Coll scaffolds especially at D3 and D5. In contrast, macrophage count for both control wounds and wounds treated with PCL scaffolds remained consistent up to D5. At D3, the average macrophage count for wounds treated with 3D PCL/Coll scaffolds was 43 ± 5 per field of view, significantly higher than the control and 3D PCL scaffold-treated wounds (*p* < 0.0001 and *p* < 0.01, respectively, Figure 3D). A similar trend was observed at D5, where wounds treated with 3D PCL/Coll scaffolds have an average of 54 ± 12 macrophages per field of view, significantly higher than the control and 3D PCL scaffold-treated wounds (*p* < 0.0001, Figure 3D).

**Figure 4:**
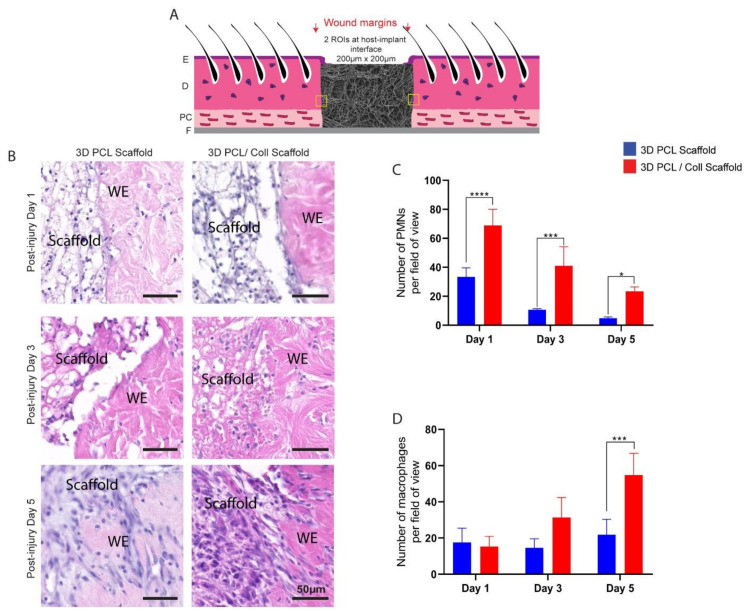
(**A**) Schematic of tissue section, where two regions of interest were cropped for quantification. E: epidermis. D: dermis. PC: panniculus carnosus. F: facia. (**B**) Representative H&E images taken from within scaffold at post injury days 1, 3 and 5. Wounds were implanted with 3D PCL scaffold or 3D PCL/Coll scaffold. WE: wound edge. Scale bar represented by black line for 50 µm. (**C**) Quantification of dermal PMNs in scaffolds. One-way ANOVA; Tukey’s post hoc test (n = 4, where n represents 1 biological repeat). *: *p* < 0.5, ***: *p* < 0.001, ****: *p* < 0.0001. (**D**) Quantification of macrophages in scaffolds. One-way ANOVA; Tukey’s post hoc test (n = 4, where n represents 1 biological repeat). ***: *p* < 0.001.

PMNs and macrophages at the host–implant interface were also counted and analysed to reveal a possible foreign body reaction towards these scaffolds (Figure 4B). Consistent with the analysis performed around the wound bed, PMN numbers reduced from both 3D PCL and 3D PCL/Coll scaffolds as time progressed. However, at all time points, the PMN count was significantly higher at 3D PCL/Coll scaffold’s interface as compared to 3D PCL scaffold’s (*p* < 0.0001 at D1, *p* < 0.001 at D3, *p* < 0.05 at D5, Figure 4C). While the macrophage count at the 3D PCL scaffold interface remained consistent up to D5, there was a massive influx of macrophages into 3D PCL/Coll scaffolds especially at D5 (55 ± 12 macrophages per field of view, *p* < 0.001, Figure 4D).

### 3.4. Accelerated Degradation of 3D PCL/Coll Scaffolds

**Figure 5:**
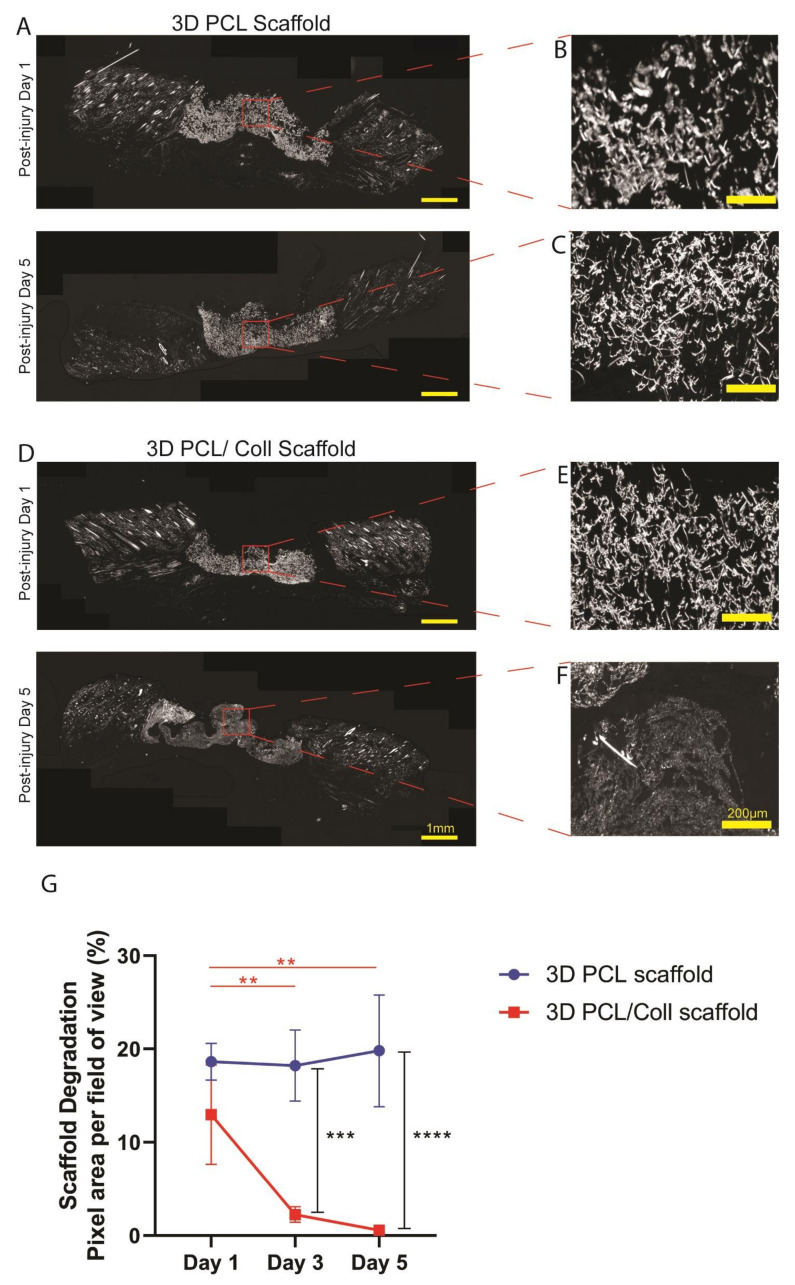
(**A**) Polarized microscopy images of tissue sections with 3D PCL scaffolds implanted at the wound bed at post injury days 1 and 5. Scale bar represented by yellow line for 1 mm. (**B**,**C**) High-power images of (**A**). Scale bar represented by yellow line for 200 µm. (**D**) Polarized microscopy images of tissue sections with 3D PCL/Coll scaffolds implanted at the wound bed. Scale bar represented by yellow line for 1 mm. (**E**,**F**) High-power images of (**D**). Scale bar represented by yellow line for 200 µm. (**G**) Quantification of scaffold degradation. One-way ANOVA; Tukey’s post hoc test (n = 4, where n represents 1 biological repeat). **: *p* < 0.01, ***: *p* < 0.001, ****: *p* < 0.0001. No significant difference in pixel area was observed from post injury day 1 to day 5 for 3D PCL scaffold (*p* = 0.9974). No significant difference in pixel area was observed between 3D PCL scaffold and 3D PCL/Coll scaffold at post injury day 1 (*p* = 0.3081).

Polarized light images of the wound sections were captured and the pixel area per field of view of the scaffolds implanted in the wound beds were analysed to investigate the structural integrity of both 3D PCL and 3D PCL/Coll scaffolds (Figure 5). The 3D PCL scaffolds remained structurally stable. No significant difference in pixel area was observed from D1 (18.6 ± 1.96%) to D5 (19.8 ± 5.99%) (Figure 5G). At D5, fibrous structure within the 3D PCL scaffolds was preserved (Figure 5A). High-power images within these scaffold regions revealed that there were minimal signs of breakdown and pores collapsing even at D5 (Figure 5B,C). On the other hand, 3D PCL/Coll scaffolds were less structurally stable, and signs of scaffold breakdown appeared at D3. A significant reduction in pixel area from D1 to D3 was observed (13.0 ± 5.33% to 2.25 ± 0.845%, *p* < 0.001, Figure 5G). By D5, from high-power images, the fibrous structure of 3D PCL/Coll scaffolds could not be detected and the pores within these scaffolds appeared to have collapsed (Figure 5F).

### 3.5. Enhanced Migration of Fibroblasts and Formation of Blood Vessels in 3D PCL Scaffolds

**Figure 6:**
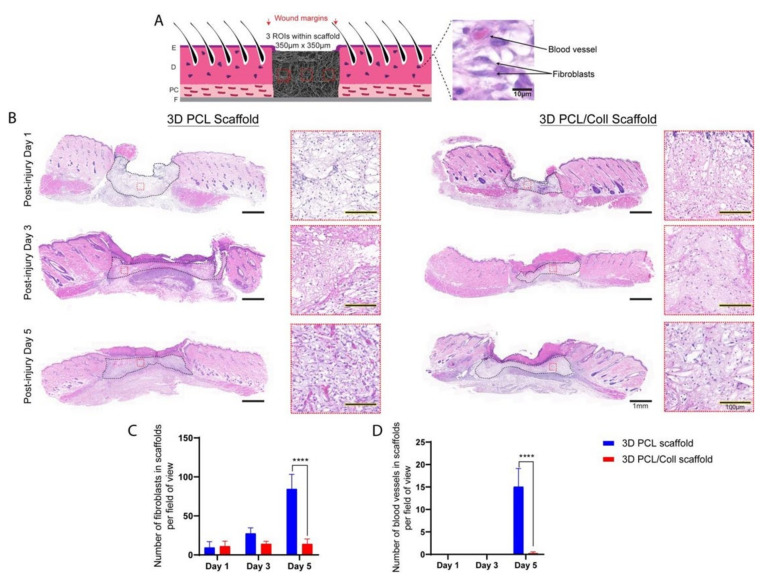
(**A**) Schematic of tissue section, where three regions of interest were cropped for quantification. E: epidermis. D: dermis. PC: panniculus carnosus. F: facia. (**B**) H&E images of wounds treated with either 3D PCL scaffold or 3D PCL/Coll scaffold across the three time points. A lower-power image illustrates the change in density within the scaffolds while higher-power images depicts the extent of fibroblast infiltration and vascularization within the scaffolds at each time point. Scale bar represented by black line for 1 mm and black line traced with yellow dotted line for 100 µm. (**C**) Quantification of fibroblasts within each field of view. One-way ANOVA; Tukey’s post hoc test (n = 4, where n represents 1 biological repeat). ****: *p* < 0.0001. (**D**) Quantification of blood vessels within each field of view. Kruskal–Wallis; Mann–Whitney test (n = 4, where n represents 1 biological repeat). ****: *p* < 0.0001.

Sister sections of the wounds stained with H&E were analysed for new ECM deposition and blood vessel formation within the scaffold region (Figure 6). The 3D PCL scaffolds appeared to have a greater degree of fibroblast infiltration at D5 compared to 3D PCL/Coll scaffolds and hence, the former appeared optically denser than the latter (Figure 6B). To understand this difference, a closer look revealed the difference in cell types populating the scaffolds—3D PCL scaffolds were populated with a majority of fibroblasts while 3D PCL/Coll scaffolds were occupied by a majority of inflammatory cells (Figure 6B). A significantly higher number of fibroblasts was observed in 3D PCL scaffolds at D5 than within the 3D PCL/Coll scaffolds (*p* < 0.0001, Figure 6C). In addition, at D5, the high-magnification view with the implanted scaffold revealed significant formation of new blood vessels within the 3D PCL scaffolds, a feature clearly lacking in the 3D PCL/Coll scaffold (*p* < 0.0001, Figure 6D).

### 3.6. Deposition of New ECM within 3D PCL Scaffolds

**Figure 7:**
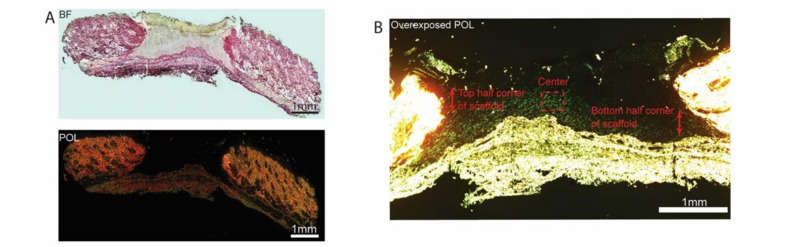

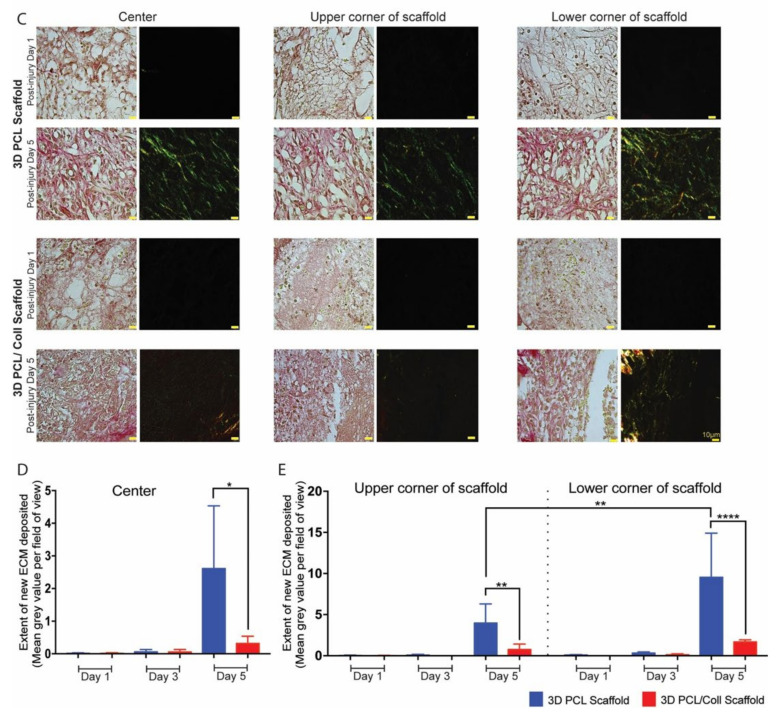
(**A**) Brightfield microscopy image (top) and polarized microscopy image of a representative tissue section stained with picrosirius red. Scale bar represented by black or white line for 1 mm. (**B**) Overexposed polarized microscopy image of scaffold implanted in wound bed to depict new collagen deposits. Scale bar represented by white line for 1 mm. (**C**) Higher-power images within the respective scaffolds to confirm the deposition of new collagen fibers along the 3D PCL scaffold’s structure. Scale bar represented by yellow line for 10 µm. (**D**) Quantification of new ECM deposition at the center of scaffold. One-way ANOVA; Tukey’s post hoc test (n = 4, where n represents 1 biological repeat). *: *p* < 0.05. (**E**) Quantification of new ECM deposition at the top and bottom half corners of scaffold. One-way ANOVA; Tukey’s post hoc test (n = 4, where n represents 1 biological repeat). **: *p* < 0.01, ****: *p* < 0.0001.

To further prove the formation of new ECM within 3D PCL scaffolds, picrosirius red (PSR) staining was performed on sister sections of the wounds and stained sections were imaged (Figure 7). Polarized light imaging revealed that fine strands of new collagen could be detected in a variety of regions. The center, lower and upper corners of scaffolds, were analysed (Figure 7B,C). Prominent green strands of collagen were observed in 3D PCL scaffolds at D5 (Figure 7C). A significantly higher mean grey value per field of view was observed in 3D PCL scaffolds at the center region (*p* < 0.05), top (*p* < 0.001) and bottom (*p* < 0.0001) half corners of these scaffolds at D5 as compared to 3D PCL/Coll scaffolds (Figure 7D,E). Notably, the bottom half corner of 3D PCL scaffolds at D5 had the highest mean grey value when compared to the other regions of the scaffold (Figure 7E, *p* < 0.001).

### 3.7. Successful Re-Epithelialization over 3D PCL Scaffolds

**Figure 8:**
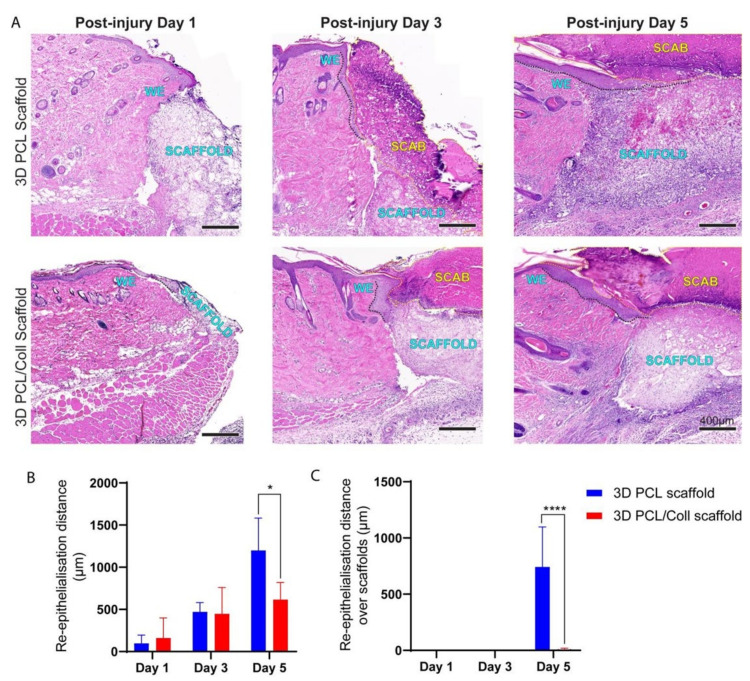
(**A**) H&E images of wound edges across all time points. Wounds were treated either with 3D PCL scaffold or 3D PCL/Coll scaffold. WE: wound edge. Black dotted line: re-epithelisation tongue. Yellow dotted line: scab. Scale bar represented by black line for 400 µm. (**B**) Quantification of re-epithelialisation distance from wound edge. One-way ANOVA; Tukey’s post hoc test (n = 4, where n represents 1 biological repeat). *: *p* < 0.05. (**C**) Quantification of re- epithelialisation over scaffold only. Kruskal–Wallis; Mann–Whitney test (n = 4, where n represents 1 biological repeat). ****: *p* < 0.0001.

H&E-stained sections were used to evaluate the extent of nascent epithelial tongue outgrowth and if re-epithelialization successfully occurred over these 3D scaffolds (Figure 8). The extent of nascent epithelial tongue outgrowth from the wound edge was significantly retarded especially at D5 for wounds treated with 3D PCL/Coll scaffolds (Figure 8A,B, *p* < 0.05) as compared to 3D PCL scaffold-treated wounds. Hyper-thickening of the leading edge hindered the migration of keratinocytes for 3D PCL/Coll scaffold-treated wounds (Figure 8A) and re-epithelialization did not occur over these scaffolds (Figure 8A and 8C). In contrast, nascent epithelial tongue successfully re-epithelialized over 3D PCL scaffolds at D5 (Figure 8A,C, *p* < 0.0001). Epithelial tongues of wounds treated with 3D PCL scaffolds were observed to have migrated out from the wound edges and thinned nicely at D3 (Figure 8A). Upon the deposition of new ECM along with the formation of new blood vessels within 3D PCL scaffolds at D5, the leading edge turned 90 degrees and keratinocytes crawled over the surface of the scaffold, forming new epidermis over these PCL scaffolds allowing them to fully integrate within the newly formed tissue (Figure 8A,C, *p* < 0.0001).

## 4. Discussion

We successfully demonstrated the feasibility of a wet electrospinning method to fabricate ECM-mimicking scaffolds that promoted fibroblast ingrowth, new ECM deposition and tissue integration with minimal inflammatory response. Existing electrospun scaffolds for wound healing are limited to collecting thin, almost two-dimensional (2D) sheet-like fiber mats on metallic collectors [15,16,17,18,19]. While some studies have reported positive clinical outcomes, these scaffolds were typically adopted as wound dressings and not as dermal substitutes [20]. In studies where the scaffolds were placed in wound beds, there is generally a lack of information on how they integrate into the host tissue at the early stages of wound healing [21,22,23,24]. Instead, most studies have examined late time points, where the scaffolds have broken down or the wounds were completely re-epithelialized [24]. Based on our previous studies, in the former scenario, an aggressive inflammatory response is likely to have broken down the scaffolds while in the latter, the scaffolds will have behaved like a scab and re-epithelialization had taken place under it. In both situations, the host–implant response fails to allow tissue integration with the implanted electrospun scaffold. With these shortcomings in mind, we adopted the wet electrospinning technique to fabricate thicker “3D” scaffolds which better mimic native ECM and examined its impact on the early stages of wound healing.

Electrospun scaffolds can modulate cellular behavior in various aspects [25]. However, cellular infiltration and growth have been limited to the densely packed 2D structures fabricated from conventional electrospinning [26]. By altering the collector, we fabricated 3D fibrous scaffolds with interconnected pore structures (Figure 1A–C) that mimicked the porous native ECM of the dermis. Instead of collecting in air and onto the metallic collector, the collection of fibers in a bath of absolute ethanol decreased the bulk density of the fiber matrix [27]. Due to its low surface tension, absolute ethanol was chosen as the liquid bath collector [9]. This low surface tension is crucial for fiber penetration and sinking into the bath, preventing fibers from floating on the liquid surface and forming a 2D fiber mat [9].

Collagen bundles are the primary structural protein that provides strength to the dermis [28]. In particular, type 1 collagen forms the majority [29]. To improve biocompatibility to the dermis, rat tail type 1 collagen was added to PCL. However, negative healing responses were observed with these 3D PCL/Coll scaffolds. In particular, an aggressive inflammatory response towards these scaffolds was observed. The ultimate goal of inflammation is to prevent infection and sepsis [30]. This process aids healing by destroying or neutralizing the pathogen and/or foreign body. However, when a foreign body is present for long periods of time, the inflammatory process is classified as chronic [31].

On close examination, both the physical structures of 3D PCL/Coll scaffolds and the surrounding tissues of these scaffolds were infiltrated with polymorphonuclear cells characteristic of neutrophils and later macrophages (Figure 3B andFigure 4B) typical of the early stages of a foreign body reaction [32]. The infiltration and accumulation of large numbers of inflammatory cells in these 3D PCL/Coll scaffolds could be responsible for their rapid breakdown (Figure 5) [33]. As these scaffolds rapidly degrade in the wound, inflammation spreads beyond the wound bed into the surrounding dermis (Figure 3B), where there is potential for the inflammatory cells to cause damage to intact tissue [33]. The presence of the 3D PCL/Coll scaffolds and/or their broken-down products could perpetuate inflammation for an extended period of time until the scaffolds are completely eradicated from the wounds [31]—trapping these wounds and their surrounding tissues within the proinflammatory phase. Such a chronic inflammatory environment is deleterious to wound healing as the accumulated leukocytes may produce excessive levels of matrix metalloproteinases (MMPs) in the presence of pro-inflammatory signals [34]. Particularly, the persistently high levels of both MMP-2 and MMP-9 degrade and decrease the bioactivity of growth factors available to support the healing of the wound. In addition, the dysregulated levels of these MMPs are known to degrade both new and existing ECM—trapping the wound in a non-healing state [34,35]. Human chronic wounds typically contain high numbers of inflammatory cells and are trapped in a chronic proinflammatory state [36]. Hence, the association of these 3D PCL/Coll scaffolds with severe inflammation could potentially be harmful.

Epidermal healing was also observed to be perturbed in wounds treated with 3D PCL/Coll. Epidermal wound edges in contact with these scaffolds formed non-migratory, thickened bulbs of keratinocytes (Figure 2B). Interestingly, this feature is seen in perturbed wound healing (e.g., human chronic wounds and wounds on diabetic rats [37,38,39,40]) and is responsible for lack of migration and the delayed closure of wounds [41]. Additionally, minimal integration of these scaffolds with the surrounding tissues was observed especially at D5. Few fibroblast-like cells were observed within these structures (Figure 6B). There was a lack of vascularization in these scaffolds as well (Figure 5B). Importantly, there was minimal new ECM deposition within these scaffolds (Figure 7C). The negative healing responses to these 3D PCL/Coll scaffolds is consistent with the observations from our previous work, where pure collagen electrospun scaffolds perturbed wound healing [8]. Here, the reduction in collagen content to 20% did not seem to reduce the negative impact on wound healing from the collagen. Conversely, in the absence of collagen, 3D PCL scaffolds successfully promoted wound healing with minimal inflammatory response. More PMNs were observed within and around these 3D PCL scaffolds at D1 (Figure 3B and Figure 4B). However, these cells were gradually lost and replaced by incoming macrophages at D3 and D5 (Figure 3C,D and Figure 4C,D). This observation is consistent with the resolution of the inflammatory phase during wound healing [42]. Furthermore, as the inflammatory phase resolved, more fibroblast cells were found within these 3D PCL scaffolds, suggesting that the ECM recovery was being stimulated. This process continued until D5, where extensive new ECM deposition and new blood vessel formation were observed in the PCL scaffolds.

Migration of keratinocytes from the wound edges of 3D PCL scaffold-treated wounds was observed. Thin epithelial tongues at these wound edges were similar to the wound edges of control wounds, where no 3D scaffolds were implanted (Figure 2B). As new ECM is deposited within these scaffolds, keratinocytes at the epithelial tongue migrated over these scaffolds (Figure 8). Successful re-epithelializationover these scaffolds was observed only after the deposition of new ECM within these physical structures. This observation is consistent with existing work, where the presence of ECM components was found to have a direct impact on keratinocyte motility [43].

Although PCL lacks cell recognition ligands [44], fibroblasts and endothelial cells were still able to infiltrate and attach within the 3D PCL scaffolds. We speculate that the hydrophobicity of PCL [45] could have favored protein adsorption. Hence, vital healing factors (e.g., growth and immune factors) via wound exudates could be trapped within the 3D PCL scaffold. Cell infiltration, proliferation and growth could have been encouraged within this physical structure due to the presence of these healing factors [46,47]. However, when adopting these scaffolds to promote chronic wound healing, wound exudates should be managed with an additional absorbent dressing. Unlike exudates from acute wounds, chronic wound exudates are plagued with high levels of proinflammatory cytokines [48]. Trapping chronic wound exudates within these scaffolds could potentially aggravate the wound healing process.

In this work, we have also evaluated the structural integrity of the wet electrospun 3D scaffolds when implanted within wounds (Figure 5). Polarized light microscopy, an imaging technique that visualizes birefringent samples [49], was adapted to understand the extent of scaffold degradation in situ. During electrospinning, the solidification of polymer fibers and organization within the polymer chains generates birefringent fibers [12,50]. Hence, the presence of these electrospun scaffolds can be visualized under polarized light microscopy. The 3D PCL scaffolds’ fibrous structures were clearly observed even at D5. In contrast, these fibrous structures could not be clearly identified in 3D PCL/Coll scaffolds. These fibrous structures could be essential in maintaining a porous matrix. A porous scaffold is crucial to support the ingrowth of blood vessels necessary for oxygen and nutrient transport [51]. In addition, porosity also plays an important role in regulating cell adhesion and migration. High porosity has been found to provide a higher surface area for cell-matrix interactions and sufficient space for ECM regeneration [52]. We speculate that these fibrous structures could have supported fibroblast attachment, encouraged blood vessel formation with the PCL scaffolds and promoted new ECM deposition.

Collagen is a commonly used material in tissue engineering. Many commercialized products designed to encourage wound healing contain collagen [53,54,55] Interestingly, this data contradicted the positive healing outcomes as reported previously by other groups [53,54,55]. The observed negative healing outcomes could be due to the instability of the 3D PCL/Coll scaffolds and not solely due to the presence of collagen. In this work, collagen was not crosslinked nor included as an additional sample group here given the consideration that stiffer matrices are known to impair the wound healing process. In particular, within stiff three-dimensional matrices, fibroblasts’ ability to spread and elongate decreases [56]. More importantly, their ability to remodel the matrix decreases with increased stiffness [56,57]. Hence, a stiff matrix will not be ideal for encouraging wound healing. However, chemical crosslinkers could be added in the future to further verify this. On the other hand, the composite of PCL and collagen, along with the wet electrospinning fabrication method, could have also altered the material chemistry, possibly activating the inflammatory response. Notably, collagen was incorporated initially with hopes of enhancing cell attachment. However, the incorporation of collagen accelerated scaffold degradation and caused the 3D PCL/Coll scaffold to collapse—possibly due to its poor integration in the wound bed. The collagen content could be reduced to less than 10% in the future to understand if the best of both worlds could be achieved, i.e., encourage cell attachment yet have a stable collagen-containing scaffold.

Of note, the degradation of PCL, in the presence of collagen, could have been accelerated—interaction of PCL polymer chains with collagen fibers could reduce the packing density of PCL polymer chains and in turn, facilitated the degradation of PCL. Although unlikely and inconclusive, the poor healing outcome observed with 3D PCL/Coll scaffolds could be partially attributed to the degradation of PCL—in general, degradation of biomaterials, at an injury site, is known to induce inflammation as a result of foreign body reaction [32].

In this work, picrosirius red (PSR) was used to investigate the deposition of new extracellular matrix within the scaffolds placed within the wound bed. Since 3D PCL/Coll scaffolds contained collagen, there could be a possibility that PSR stained the collagen content within the scaffold. However, the likelihood of staining collagen within these scaffolds is low especially since a low concentration of collagen was added. Moreover, the collagen was not crosslinked and hence, could have been degraded by day 3.

Currently, there are no FDA-approved therapeutic eluting wound care materials. Although there are many groups working on therapeutic delivery systems that aim to promote wound healing, most of this work focuses on developing dressings but not dermal substitutes [35]. Here, we demonstrated the potential of 3D PCL scaffolds in promoting tissue integration and re-epithelialization with minimal inflammatory response. These scaffolds can be further developed into therapeutic delivery systems that supply wound healing agents either through passive delivery mechanisms (e.g., diffusion of therapeutics from the surface of a material or through material breakdown) or active delivery mechanisms (e.g., stimuli-triggered release of therapeutics).

Through this work, we discovered the advantages of wet electrospinning in fabricating 3D scaffolds that promote full-thickness wound healing. In particular, the promising outcomes observed at early time points of healing suggests that the 3D PCL scaffold holds tremendous potential for chronic wound treatment. PCL is known to have long degradation time and could take up to 2 years to degrade [58]. Hence, to confirm these positive healing outcomes we observed at these early time points, more experiments to evaluate long-term integration, ECM remodeling, and scar formation will be conducted. Importantly, the extended presence of the 3D PCL scaffolds must be evaluated as the wound heals. In addition, more in-depth studies on other delayed wound healing models in both rodents and pigs will be explored. These scaffolds will also be tested on human chronic wound samples to explore the positive healing outcomes observed in this study. Additional physical characterisation studies (e.g., porosity, mechanical strength and swelling ratio) on these wet electrospun scaffolds could also be conducted in the future to better understand other physical properties of such scaffolds that encourage wound healing—in hopes of discovering and developing artificial dermal substitutes.

## 5. Conclusions

Taken together, we demonstrated the potential of 3D PCL scaffolds for promoting wound healing. The attenuated inflammatory response towards these scaffolds was coupled with enhanced fibroblast migration and more vascularization within these scaffolds. Of particular note, upon the deposition of new ECM in the scaffolds, keratinocytes successfully migrated over the scaffolds. Our observations have further confirmed and emphasized the importance of ECM deposition within dermal substitutes to encourage re-epithelialization over scaffolds, integration into the wound and the eventual wound closure.

## Data Availability

The data presented in this study are available on request from the corresponding author.

## Supplementary Materials

The following supporting information can be downloaded at: https://www.mdpi.com/article/10.3390/bioengineering9070324/s1, Figure S1: (**A**) Schematic of wet electrospinning setup. (**B**) Types of scaffold implanted into the wounds on the back of the rat. Placement of scaffolds and Pluronic gel were randomised from rat to rat.
