# Supplementary material for: Bio-Mimicking Acellular Wet Electrospun Scaffolds Promote Accelerated Integration and Re-Epithelialization of Full-Thickness Dermal Wounds
Source: Bioengineering (Basel). 2022 Jul 18;9(7):324. doi: 10.3390/bioengineering9070324 (PMC9312172; doi:10.3390/bioengineering9070324)
Supplement: Supplementary file 1 [file bioengineering-09-00324-s001.zip › bioengineering-1675615-supplementary.pdf]

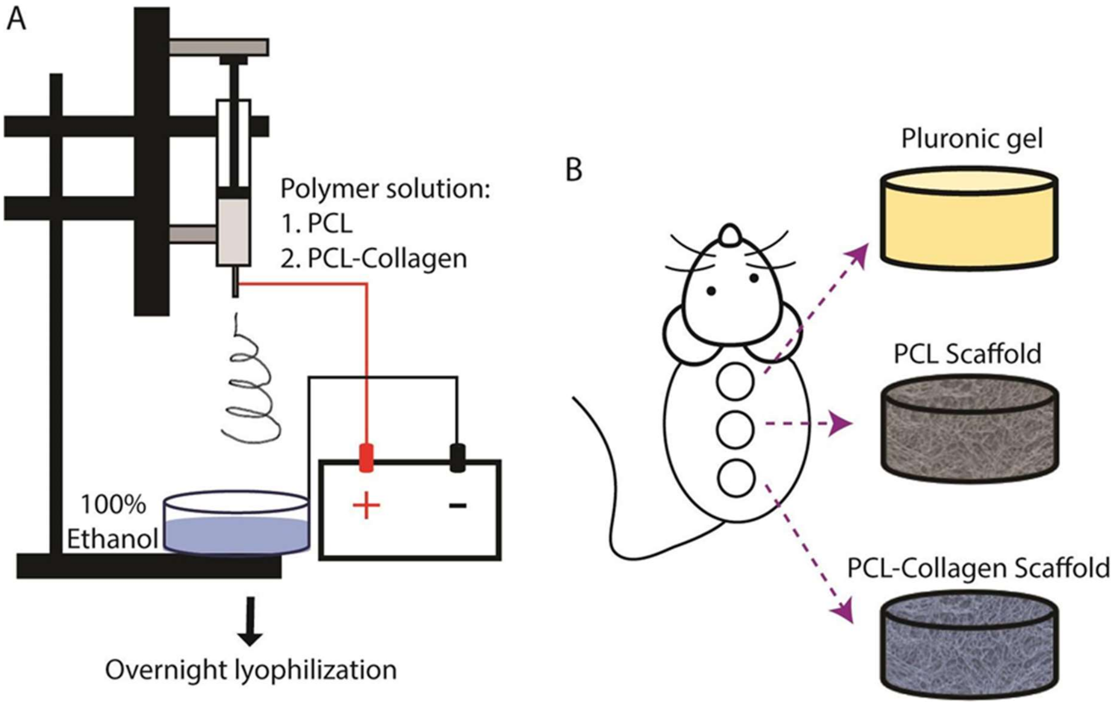

Supplementary Figure S1. (A) Schematic of wet electrospinning setup. (B) Types of scaffold implanted into the wounds on the back of the rat. Placement of scaffolds and Pluronic gel were randomised from rat to rat.
